# Weight-Bearing Estimation for Cane Users by Using Onboard Sensors

**DOI:** 10.3390/s19030509

**Published:** 2019-01-26

**Authors:** Joaquin Ballesteros, Alberto Tudela, Juan Rafael Caro-Romero, Cristina Urdiales

**Affiliations:** 1 Division of Intelligent Future Technologies, Mälardalen University, 721 23 Västerås, Sweden; 2 Department of Electronic Technology, University of Malaga, 29071 Malaga, Spain; ajtudela@uma.es (A.T.); jrcaro@uma.es (J.R.C.-R.); acurdiales@uma.es (C.U.)

**Keywords:** smart cane, weight-bearing, gait analysis, health monitoring

## Abstract

Mobility is a fundamental requirement for a healthy, active lifestyle. Gait analysis is widely acknowledged as a clinically useful tool for identifying problems with mobility, as identifying abnormalities within the gait profile is essential to correct them via training, drugs, or surgical intervention. However, continuous gait analysis is difficult to achieve due to technical limitations, namely the need for specific hardware and constraints on time and test environment to acquire reliable data. Wearables may provide a solution if users carry them most of the time they are walking. We propose to add sensors to walking canes to assess user’s mobility. Canes are frequently used by people who cannot completely support their own weight due to pain or balance issues. Furthermore, in absence of neurological disorders, the load on the cane is correlated with the user condition. Sensorized canes already exist, but often rely on expensive sensors and major device modifications are required. Thus, the number of potential users is severely limited. In this work, we propose an affordable module for load monitoring so that it can be widely used as a screening tool. The main advantages of our module are: (i) it can be deployed in any standard cane with minimal changes that do not affect ergonomics; (ii) it can be used every day, anywhere for long-term monitoring. We have validated our prototype with 10 different elderly volunteers that required a cane to walk, either for balance or partial weight bearing. Volunteers were asked to complete a 10 m test and, then, to move freely for an extra minute. The load peaks on the cane, corresponding to maximum support instants during the gait cycle, were measured while they moved. For validation, we calculated their gait speed using a chronometer during the 10 m test, as it is reportedly related to their condition. The correlation between speed (condition) and load results proves that our module provides meaningful information for screening. In conclusion, our module monitors support in a continuous, unsupervised, nonintrusive way during users’ daily routines, plus only mechanical adjustment (cane height) is needed to change from one user to another.

## 1. Introduction

A 2017 United Nations report on world population ageing [1] shows that the number of persons over 60 years in 2050 will double with respect to 2017, i.e., in 2050 one out of five people worldwide will be seniors. Indeed, population ageing is already significant in Europe and Northern America, where currently more than one out of five persons are already over 60. Hence, healthy ageing has become the main concern. One of the main tools to promote healthy ageing is, reportedly, monitoring and feedback to users [2], especially for the most vulnerable population, like persons with some degree of disability. Mobility monitoring has attracted major interest, as it is fundamental to keep a healthy and active lifestyle and to remain autonomous. The simpler, most popular approach to mobility monitoring are activity/fitness trackers, that provide data on distance walked or run, calorie consumption, and, in some cases heartbeat or quality of sleep. However, parameters like distance walked are only indirectly related to a person’s condition. Alternatively, gait (and posture) analysis is widely acknowledged as a clinically useful tool for identifying problems with mobility. Poor gait may lead to musculoskeletal pain in different parts of the body, whereas a good gait will reduce the risk of joint problems, help good recovery after injury and/or surgery, improve mobility in the elderly and reduce fall risk [3]. Consequently, gait monitoring has attracted considerable attention.

Clinically, gait analysis is performed via scales like the Tinetti Mobility Test [4]: experts observe patients performing different tasks and manually provide scores for several indicators. However, this process is slow and requires significant time from patient and clinician. There are technology-based alternatives to this approach, like using force/pressure sensors on a walking surface like a treadmill or a walkway [5,6] or optical motion capture systems [7,8,9,10,11,12]. However, people need to be assessed in specific installations, and experts are often needed to set and/or calibrate equipment. Gait assessment during Activities of Daily Living (ADLs) may be achieved using wearable sensors, e.g., sole pressure sensors [13,14] and/or inertial sensors [15,16,17]. However, some of these wearables may not be comfortable nor easy to attach/calibrate [18] so people often do not carry them. Sensors can instead be attached to mobility aids if people require them for their ADLs [19,20]. Indeed, smart wheelchairs and rollators often include different sensors. However, attaching them to canes, the most spread mobility aid, with minor alteration of their ergonomics (Modifications that affect ergonomics or center of gravity in walking aids require full analysis, validation, and standardization of the modified device, a process usually costly and slow) is usually harder.

Load on a cane can be used to estimate a person’s partial weight-bearing by calculating the differences between the users’ weight and the load. Partial weight-bearing provides information about how much load the user supports on their affected side. This is an indirect measure of their condition and it can be used to monitor their evolution, provide feedback and, in clinical treatments, to adapt their rehabilitation process [21]. There are different solutions to measure load on a cane. In specific, fixed environments, a walkway with a dense matrix of force sensors can be used, e.g., Strideway™ (Strideway System; Tekscan, Inc., South Boston, MA, USA) (Strideway System. Available online at: https://www.tekscan.com/products-solutions/systems/strideway-system?tab=applications). These systems measure weight-bearing accurately and, hence, the cane load by default. However, they only measure a very limited number of meters on a straight line, plus they are expensive and, as aforementioned, constrained to specific environments. Hence, this work focuses uniquely on onboard cane sensors. Currently, onboard sensors on most smart canes are located either on the handgrip [22,23], shaft [23,24,25,26,27] or tip [22,24]. Every location has advantages and disadvantages. Placing sensors on the handgrip or tip may involve major cane modifications [22,23]. As both locations affect how users support their weight, these modifications must be ergonomic. Unfortunately, granting ergonomics requires an extensively validation process. On the other hand, the shaft allows more space to place the electronic. However, this approach may involve changes in the cane center of gravity and, in its weight [23,24,25,27].

This work proposes an affordable add-on module for long-term monitoring of load on cane. The obtained value can be used for long-term assessment of users’ condition, either for preventive healthcare or for evaluation of degenerative or rehabilitation processes. The module has been designed to be extensively available to as many potential users as possible, so its main goals are: (i) compatibility with existing commercial canes; (ii) to avoid any impact on cane ergonomics; (iii) to allow continuous, long-term use; and (iv) to keep the global cost as low as possible. Additionally, we plan to release the proposed system under a Creative Commons License to boost its reach and impact. Section 2 describes the proposed system, i.e., mechanical design and electronics. The load cane estimation is described in Section 3. Section 4 describes our methodology. Tests to validate that the module provides meaningful load estimation are presented in Section 5. Section 6 discusses our results. Finally, Section 7 presents the conclusions and future work.

## 2. Cane Adaptation

The main goal of this work is to design and develop a low-cost module that can be attached to commercial canes for load monitoring in a (fairly) simple way. Hence, we have the following specific subgoals: (i) use low-cost components; (ii) preserve cane ergonomics; and (iii) low power consumption. This is achieved through goal-oriented mechanical and electronic design.

### 2.1. Mechanical Design

**Figure 1:**
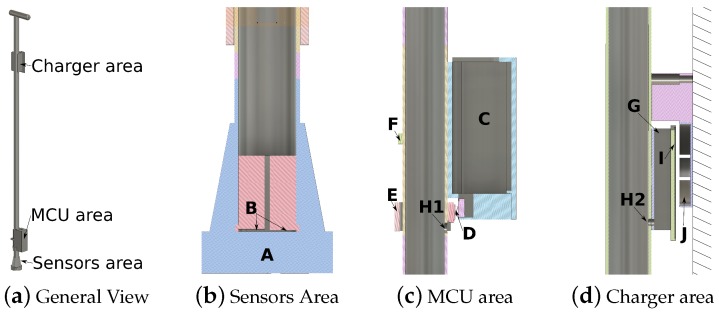
Mechanical design overview (**a**) and its parts (**b**–**d**): (A) rubber tip; (B) force sensors locations; (C) microcontroller (MCU) box; (D) connectors between charger-sensors and MCU; (E) connector ring; (F) clamp for MCU box; (G) battery and charger box; (H1) hole to wires from charger and sensors areas to MCU box; (H2) hole to wires from MCU box to charger area; (I) charging receiver location; and (J) charging transmitter location.

Our module includes three different areas: sensors area, microcontroller area and charger area (Figure 1a). The sensors have been embedded inside the shaft, as close to the tip as possible at two different depths: 0.2 mm and 1 mm via a specially designed 3D printed plastic piece (Figure 1b). Thus, the tip and handgrip designs are not modified, and we preserve their original ergonomic properties. Placing electronics inside the shaft is actually much harder and makes access difficult, so they are externally attached. Their location has been split into two different areas to minimize changes to the center of gravity of the cane: the microcontroller (MCU) area (Figure 1c) and the charging area (Figure 1d). To connect the microcontroller (MCU) with the charger and with the sensor areas, only two holes are required in the shaft (H1 and H2 in Figure 1c,d). Hence, attaching the module only involves minor cane modifications.

The charging area has been designed to avoid connectors, so elderly people with visual impairment can use it in a straight, simple way. It relies on a Qi interface and it is divided into two different parts. The first part is a fixed piece that can be attached to a wall (**J** in Figure 1d). The second one is a flat piece attached to the shaft cane (**I** in Figure 1d). The Qi interface uses inductive charging over close distances. Hence, users do not need to plug any connector; they just need to support the cane on the wall piece and the battery starts charging. Each piece has been modelled using Autodesk^®^ Fusion 360 and it has been 3D printed with Ultimaker2 using PLA plastic (All 3D models are freely available online at https://github.com/joaquinballesteros/Smart-Cane).

### 2.2. Electronic Design

There are different approaches to measure load on a cane. The most usual ones rely on force sensors on handgrip [22,23] and/or force sensors replacing part of the shaft [27]. Some of these solutions may affect the center of gravity and/or ergonomics of the cane depending on the sensor location, as commented. Nevertheless, the main drawback of most existing approaches is that they rely on relatively expensive electronic components, such as piezoelectric quartz force link with in-line amplifier (Kistler Instrument Corp., Novi, MI, USA) [27] or array of force sensors (FSR 402, Interlink Electronics, Los Angeles, CA, USA) [22], reducing affordability for end-users. Additionally, solutions do not deal with the charging process, which can be a challenge for users with vision and/or cognitive impairments.

**Figure 2:**
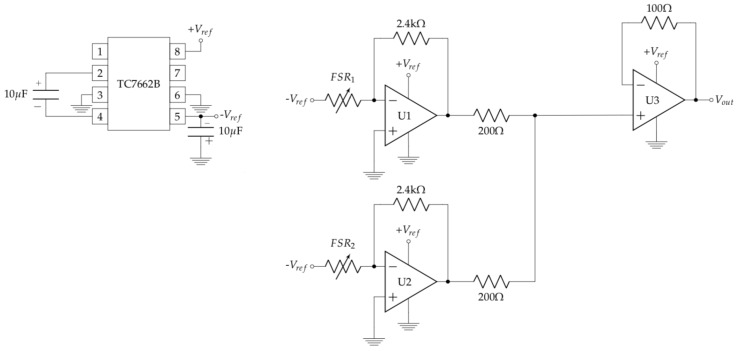
Sensor electronic board. The input signals to FS402 sensors are inverted. U1, U2 filter the force sensors outputs. Then, U3 sums both signal to obtain the output.

The main problem with force sensors is that their price increases significantly with their measurement range. Our proposed solution relies on combining two cheap force sensors at different depths into a single piece (**B** in Figure 1b) to increase their original range (see next section). We have designed a specific plastic piece to arrange them perfectly inside the shaft using a 3D printer. This approach is affordable because it only involves: two low-cost sensors (FSR 402, Interlink Electronics, Los Angeles, CA, USA), an inverter (TC7662B, Microchip Technology, Chandler, AZ, USA), an array of operational amplifiers (OPA347, Texas Instruments, Dallas, TX, USA) and some resistors and capacitors. Figure 2 shows the sensor board. The input signals to FS402 are inverted $-V_{ref}$ using TC7662B circuit. The operational amplifiers U1, U2, and U3 are adjusted with a 2.4 kΩ and 100 Ω resistors respectively to obtain the higher range of support measurement. The filtered output of FSR 402 force sensors U1, U2 are added to obtain the combination of both sensors U3. The sensor board provides a 50 Hz output.

We work with a BLE nano v2 microcontroller (nrf51822, Nordic Semiconductor, Norway). It transmits packets of 20 Bytes (8 readings) at 6.25 Hz to a paired device. Long-term monitoring requires a working plan for at least 12 h without recharging. For this reason, we have chosen a standard Qi Wireless Charger receiver with a Lipo charger (TP4056, Nanjing Top Power ASIC Corp, Nankin, China) connected to a 1S1P 500 mAh Lipo battery that reportedly provides over 4 days of use without recharging.

To heuristically calculate the system battery working life, we have performed 10 runs of the same test, consisting of feeding the cane non-stop until communication with the microcontroller is cut off, i.e., a paired device does not receive packets any longer. The battery working life is inversely proportional to the load, i.e., higher loads will lead to shorter battery life. Hence, we have used a static weight on the cane to simulate the user’s load. The period of loading versus non-loading and the amount of loaded weight have been mechanically changed to simulate a large variety of users’ behaviors. After several tests, the range battery working life turned out to be 82–89 h, i.e., more than 3 days of continuous monitoring.

Overall, the cost of the proposed system is cheap when compared to other solutions (less than USD 100 in total), plus it requires only minor modifications in the cane.

## 3. Dynamic Weight-Bearing Calculation

Canes can be used to reduce pain in one lower extremity by allowing users to support some weight on them. Load on cane depends on the person’s own weight, but also on their condition. To design a cane sensor, it is necessary to grant that its range is wide enough to capture this weight variations. As commented, low-cost sensors have lower ranges and accuracies. However, in this section we propose a method to increase their range by combining two cheap sensors into a single sensing module.

Cane users can be categorized into contralateral and ipsilateral. Users who support weight on the cane and on the closest foot to the cane at the same time are ipsilateral. The others are contralateral. Ipsilateral users reportedly load up to 7% of their body weight on the cane. Contralateral users are more frequent and more critical, as they load up to 9% [28]. These percentages may increase when users have some physical issues. Our solution relies on cheap FSR 402 force sensors to measure loads. FSR 402 sensors have a limited range up to only 10 kg. Therefore, contralateral users could weigh as much as 111 kg, whose 9% is 10 kg, before sensors saturate. Unfortunately, this limit could be significantly more restricting for users with physical issues.

**Figure 3:**
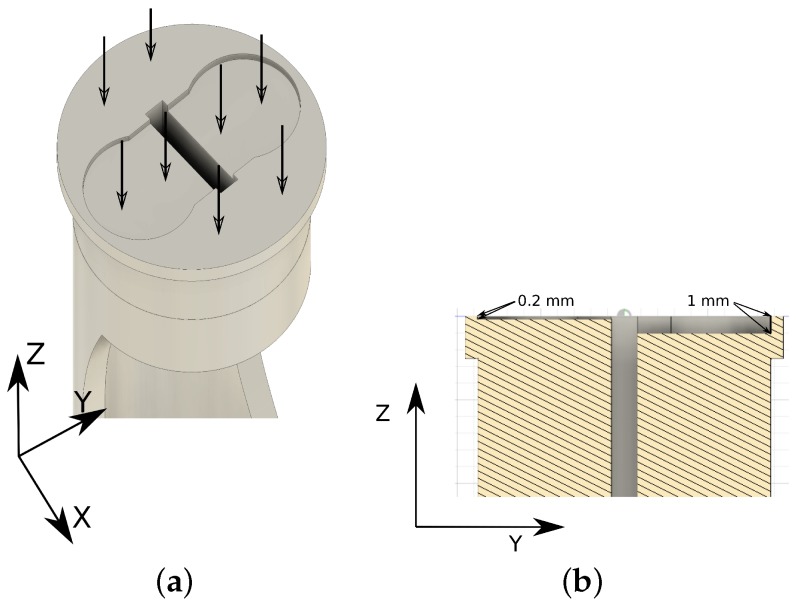
Pressure distribution is done at two different levels: (**a**) First the rubber tip pressure distribution among the force sensors and the sensor area; (**b**) Second, pressure distribution on the sensors depends on the depth.

To increase the measurement range while keeping sensors affordable, we have placed two (Due to the limit in the inner cane diameter (maximum of 22 mm), only two force sensors can be placed) FSR 402 sensors at different depths on a circular 3D printed plastic piece. First, weight is distributed all over the surface of the piece (Figure 3a), so each sensor only receives part of the load, effectively increasing the global piece range. Additionally, the tip rubber applies different pressure on each sensor (Figure 3b), i.e., lower loads will not affect the deepest sensor. Unfortunately, factors like the nature of materials, non-linearity in sensors, and other physical variables, make unfordable to analytically calculate the sensor area output function. However, it is possible to estimate it. To obtain an estimated function of the sensors’ outputs, we have calibrated the system through extensive testing using depths ranging from 0 mm to 5 mm.

**Figure 4:**
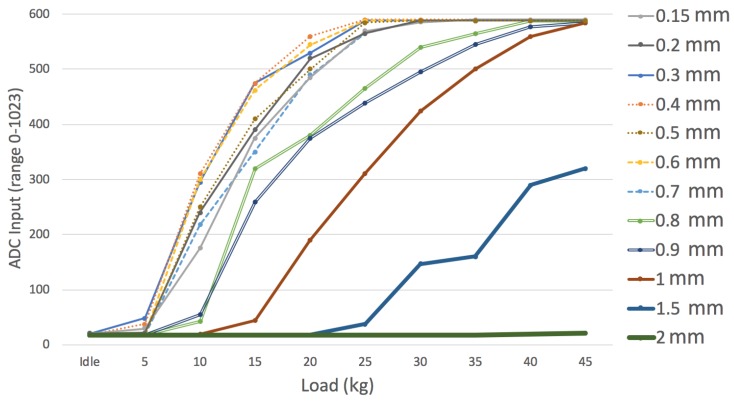
Sensor calibration for different sensor depths. Different static loads have been measured using the sensor electronic board (Figure 2) with one FSR 402 connected (the other one was bypassed).

Calibration consisted of automatically applying different static weights on the fully vertical cane when one FSR 402 was connected to the sensor electronic board (Figure 2) and the other was bypassed. The readings were performed using one Analog Digital Converter (ADC), range 0–1023, from the BLE nano v2 board. First, we tested different sensor depths to select the most appropriate one, i.e., depths that provide the best reading range. Figure 4 shows different depths where FSR 402 were located and, it shows the average load (kg) in those locations. It can be observed that deepest locations (1 mm, 1.5 mm or 2 mm) have a problem to detect lower loads as we commented previously. On the other hand, outer locations saturate the signal when higher load values are applied (0.15 mm, 0.2 mm or 0.3 mm). For these reasons, we selected 0.2 mm and 1 mm as a combination that deals successfully with low and high load on cane simultaneously.

**Figure 5:**
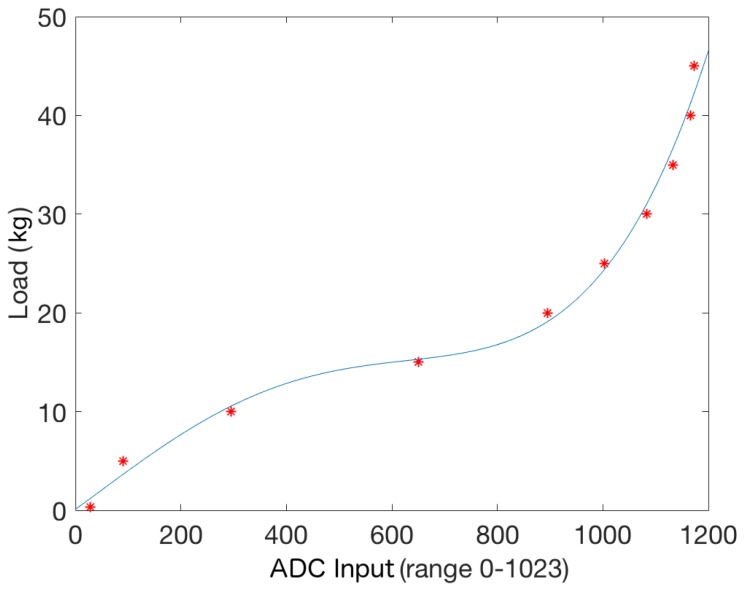
This figure shows the relation between the ADC connected to the sensor electronic board (Figure 2) and the load on the cane in kilograms when only one sensor is connected (the other one is bypassed). A 4th order polynomial has been used to estimate that transformation: $8.74\cdot 10^{-11}x^{4}-1.21\cdot 10^{-7}x^{3}+2.11\cdot 10^{-5}x^{2}+3.61\cdot 10^{-2}x+0.5191$. The mean squared error on the estimation is 0.0515 kg.

Once both locations were selected (0.2 mm and 1 mm), we applied the same procedure to find the relation between applied weights and corresponding sensors readings, now with both FSR 402 connected. Figure 5 shows the obtained relation. It can be observed that readings below 2.5 kg present a bigger mean squared error (0.1389) than measurements over this value (0.0323). This is an effect of the rubber tip pressure distribution, as low weights only affect—partially—the outermost sensor. The obtained curve can be approximated by the following quadratic equation: $8.74\cdot 10^{-11}x^{4}-1.21\cdot 10^{-7}x^{3}+2.11\cdot 10^{-5}x^{2}+3.61\cdot 10^{-2}x+0.5191$.

The designed 2-sensors piece output range grows up to 45 kg (hardware reading equal to 1023) for the best depth difference. This means that a healthy contralateral user could weigh as much as 500 kg. Even though persons with disabilities support significantly more than 9% on the cane, this upper bound is high enough for most cases.

## 4. Methodology

**Figure 6:**
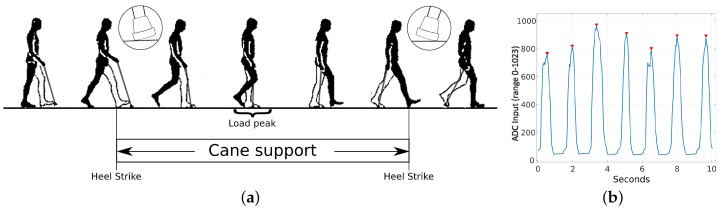
Contralateral user walking: (**a**) User walking in the support cane period; and (**b**) Input signal from force sensor during a 10 s interval in user 8 (range is 0−1023). Maximum peaks (corresponding to cane vertical position) are marked in ▾.

While users walk, the weight they bear on the cane keeps changing. To calculate the maximum support that a given user needs while walking, gait cycles can be analyzed using the adapted cane. Figure 6a shows the typical cane movements for contralateral users while they walk. During a gait cycle, maximum force is applied on the cane when it is fully vertical, as the load vector is orthogonal to the force sensors plane. The cane support cycle corresponds to the elapsed time from a heel strike of the opposite leg, with respect to the cane, to the next one. As expected, force sensors outputs fluctuate while users change their load on the cane (Figure 6b). As commented, maximum peak values correspond to the cane in a vertical position. A peak during a cane support period represents the upper bound support that a given user needs in each step. A sequence of peaks in time provides continuous information about the user load on the cane. We have used the *findpeaks* MATLAB R2016b (The MathWorks, Inc., Natick, MA, USA) function to detect all peaks during a given test. Users load on the cane during the affected leg step, but then they move the cane. The minimum time between two cane supports depends on the users’ step times. More than 99,99% of the elderly population reported a value above 0.5 s [29]. For these reasons, the *findpeaks* function has been set with a *MinPeakDistance* parameter equal to 25 (0.5 s). An additional function parameter, the *MinPeakHeight*, has been defined. It is used to filter out spurious peaks from the input signal. Its value depends on users’ load, so we have empirically set it to the average of the input signal exercise.

Our tests were carried out in two senior centers in Cordoba, Spain. As commented, we chose volunteers who require a cane for mobility. We chose to select people with different disabilities that required the cane either for balance or to reduce pain in limbs after surgery or due to degenerative conditions. We also chose people who favor, left, right, or no particular side when walking. Thus, we can check how the module responds to different load bearing profiles. In the end, we selected 10 volunteers: 8 men and 2 women. An additional participant who was initially eligible was excluded from the study, as the module showed that she did not load any weight while she walked, i.e., did not require weight support assistance. Participants were on average 83.7 years old (range 74–94 years). Table 1 shows their ages, genders, average gait speeds, and their physical diseases.

**Figure 7:**
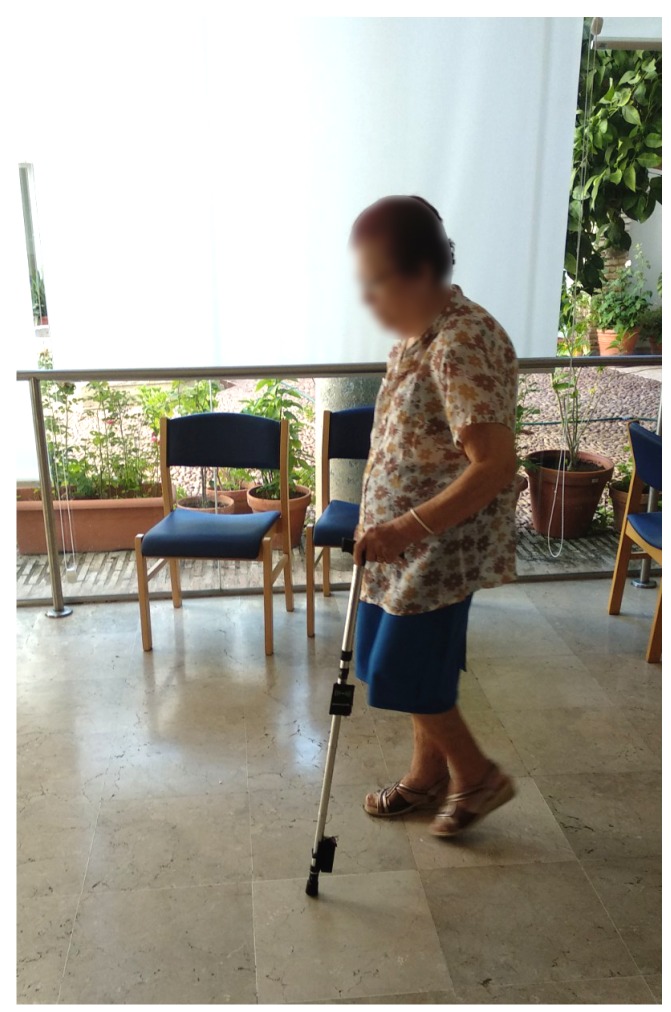
Cane user walking during a free walking test.

Volunteers were asked to complete two different popular clinical tests sequentially. All cane data was collected by a mobile phone paired with the cane via BT. First, volunteers performed the 10 m test (Figure 7). Then, they kept walking during 1 more minute approximately, depending on their condition. All tests were approved by the University of Malaga Institutional Ethical Committee and by the senior centers. Also, all volunteers signed an informed consent.

## 5. Experiments and Results

The target of the present work is to develop a low-cost, long-term module to estimate load on a cane while users walk. Hence, to validate the system, it is necessary to check that the readings of the sensor depend on the load on the cane during the gait cycle. It is important to test the device with its target population, as healthy volunteers reportedly use walking aids in different, non-consistent ways even when they try to simulate pathological gaits. Our test population includes only people who rely on a traditional cane for mobility in their ADLs (Figure 7).

We had already tested that the cane reliably measures static weights during the calibration process (Figure 4). The error was bigger when the load was below 2.5 kg (mean squared error of 0.1389). However, it is necessary to check how the module behaves during a gait cycle, with dynamically changing loads. As commented, the load on the cane depends not only on user weight but also on their condition. Hence, it is interesting to test the system with people with different conditions to check that the module works well for different gait abnormalities. Unfortunately, although weight can be normalized or accounted for, major load variations due to the condition cannot be quantified, i.e., we cannot directly predict how much weight a given person is going to support on the cane at each time instant to check whether our module is accurate or not. To correlate our readings with the condition, we assess users via their gait speed, which has been consistently reported as a meaningful parameter. We have measured manually the gait speed using two markers in the floor and a highly precise chronometer.

**Figure 8:**
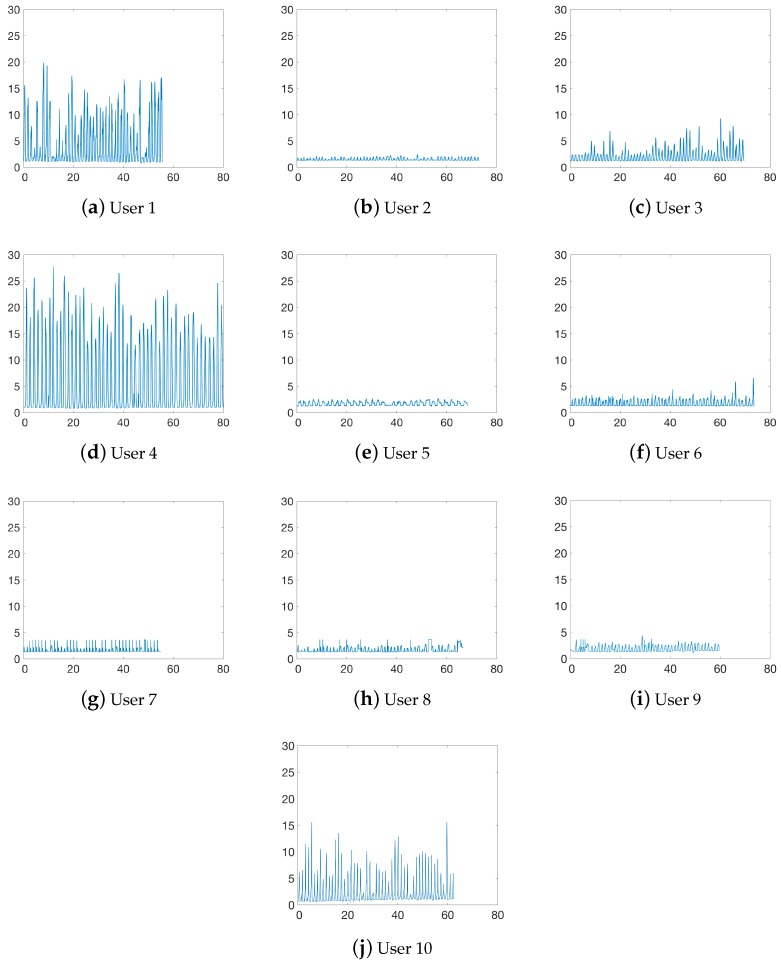
Load on cane *y*-axis (kg) over time *x*-axis (seconds). Users were suggested to walk for one minute, some of them (users 1 and 7) walked less than the minute and others more than the minute.

Figure 8 shows the cane load over time for each user presented in Table 1. We can observe that some users load significantly more weight on the cane with respect to others. For example, users 1 and 4 present peak values of 8.55 kg and 11.78 kg on average when compared to users like 2 or 5 (0.18 kg and 0.32 kg, respectively). The main reason for this variability is that, as commented, load depends largely on the users’ condition, even more than on the users’ weight, i.e., users with poor condition need more assistance. We can also notice that some users increase the load on the cane the longer they walk, like user 3 (meniscus surgery in both knees).

**Figure 9:**
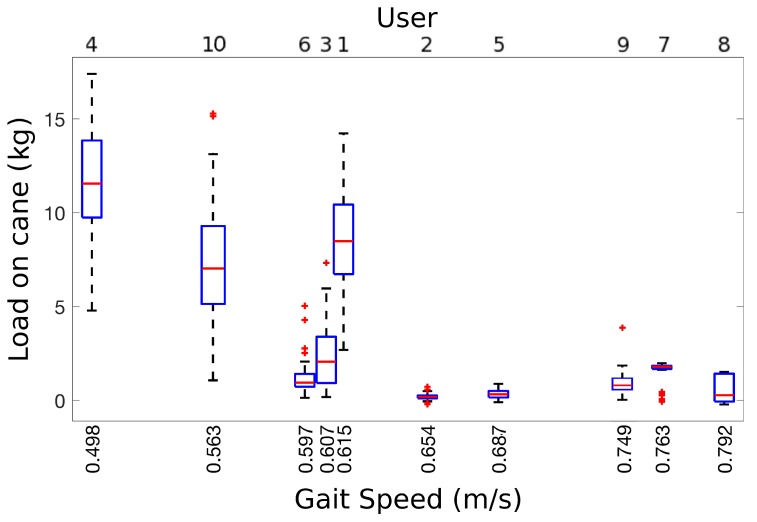
Load on cane vs users’ gait speed.

As weight bearing depends largely on condition, we had no benchmark function to determine how much weight each volunteer loaded on the cane at each time instant. Hence, we used gait speed as an indirect measure of disability to check that users with poorer conditions returned larger loads on the cane. People with severe dependencies typically present gait speeds below 0.6 m/s [30] and they are expected to bear more weight on the cane. Figure 9 shows the relationship between gait speed during our 10 m tests and the load peaks for each of our volunteers. As expected, gait speeds below 0.615 m/s are related with higher loads on the cane: 7.47 kg on average (ranging from 1.15 kg to 17.13 kg); whereas volunteers with gait speeds above its have an average load on the cane of 0.89 kg (ranging from 0.18 kg to 1.45 kg). Additionally, load variances for gait speeds under 0.615 m/s (variation range from 0.84 kg to 2.69 kg) are higher when compared to gait speeds above this limit (variation range from 0.17 kg to 0.68 kg). This gait speed relation with load on cane confirms that users with poor condition need more assistance than others with lower condition and that the need for aid depends largely on their condition. Specifically, we obtained the Pearson correlation coefficient between gait speed and median load for our volunteers ($H_{0}$ is a correlation equal or greater than 0). The resulting coefficient to −0.7473 (*p*-value 0.0065), meaning that gait speed and load are inversely related, as reported by clinicians [31]. Volunteer 6 is an outlier in this analysis because he presents a vestibular disorder, i.e., he uses the cane for balance rather than for weight bearing. If we remove him from the correlation, the coefficient is equal to −0.7971 (*p*-value 0.0050).

Finally, we can observe in Figure 8 that the upper bound for our test volunteers is equal to 29 kg. This limit fits well the target 3-days of use without charge for 8 h per day of loading. In conclusion, the proposed module meets the required constrains: (i) it is cheap and easy to add to a commercial cane; (ii) it reliably estimates load on the cane in static and dynamic conditions; and (iii) it can be used for long-term monitoring.

## 6. Discussion

The target of this work was to present a low-cost device that can be attached to commercial canes for long-term load monitoring, so it can be used for condition assessment. The proposed device has been built and tested with its target users. Results have proven that indeed: (i) it provides continuous load monitoring; (ii) it does not affect cane ergonomics; and (iii) battery allows long-term use without recharging. Additionally, results show that the load on the cane is correlated with gait speed, which is a clinically reported condition estimator.

Tests were carried out in two senior facilities in Cordoba, Andalusia. Selected volunteers required a cane for mobility. Thus, we could check load estimation when the system operates under dynamic loads through different pathological gait cycles. We also obtained users’ gait speed to check that estimated load is related to speed, i.e., condition. We did not notice any difference in users’ gait when they were using the modified cane, nor they reported any change. Hence, we validated that changes in ergonomics were not significant. Also, we did not need to recharge the cane at all for the whole test duration, so we also validated that it can be continuously used for long-term monitoring. Load results were coherent with the hypothesis: people with more significant disabilities consistently bear more weight on their cane and walk slower. The device provides dynamic loads during the gait cycle. We checked that load variation was larger for people in poorer condition and that they increased their load on the cane when they started getting tired.

The proposed system is not as reliable as other load estimation devices, like treadmills or pressure plates. However, those systems are constrained to specific installations, so they do not provide information while users perform their ADLs. Our module has three main advantages when compared to similar cane-based systems. First, it is a low-cost add-on module that can be easily attached to any commercial cane, rather than a stand-alone system that may present significantly higher costs and lower availability. The module has been designed to avoid changes in ergonomics and/or center of gravity of the original cane. Second, we have designed a novel sensor module that combines two low-cost load sensors at different depths into a single piece to increase their range. Optimal depth differences have been heuristically estimated to provide the best results even under extreme loads (up to 45 kg fully supported on the cane). Third, the system has been extensively tested under different loads to grant at least 3 days of operation without battery recharge, including power required by the communication module. Information can be gathered using any BT-equipped portable device like a smartphone or a tablet. We rely on an induction battery recharge system to facilitate its use to people with visual impairment. The measured error under static load is under 0.14 kg, which is suitable for the average load that our target population bears on their canes.

We expect that a significant number of these modules can be produced at a reduced cost to monitor specific target groups for long-term clinical gait analysis in hospital facilities and/or care centers. Unlike other equipment, the proposed system may be carried by users anywhere, to outside and inside environments, for days, so it could provide meaningful information about behavior trends and condition changes in people with specific disability profiles.

## 7. Conclusions and Future Work

This paper has presented a low-cost modular system to measure load on a cane for people with disabilities. The main goal of our work was to design an affordable prototype that could be easily attached to commercial canes and used by a wide population for long-term gait analysis. For this purpose, the system has been shared under an open license. All 3D models, plus the microcontroller software are freely available online under a Creative Commons Attribution 4.0.

The system has been tested in two senior facilities in Cordoba, Andalusia. All volunteers were elderly people that required a cane for everyday mobility and presented different disabilities. As load information is related to user’s condition, it has significant clinical value. Load analysis provided by our system may help to monitor the evolution of people after surgery, during rehabilitation or suffering degenerative processes to correct their gait, change treatments or prevent accidents like falls.

Future work will focus on two different aspects: improving the module design and extracting more information from gathered data. The module can be improved by increasing battery life and reducing BT pairing time. We plan to induce a low power consumption stage when no loads are detected for a significant period of time. Additionally, the load on peak estimation will be processed and stored on the microcontroller to reduce communication time between the microcontroller and the mobile phone. Our preliminary tests also point out that further clinically relevant information can be extracted from the system, so we will evaluate parameters of interest in gait analysis and check how many we can extract either from the current module or from an improved version. Finally, we plan to perform exhaustive testing by the target population, including usability tests to assess acceptance.

## Figures and Tables

**Table 1:** Condition and characteristics per users.

Id	Age	Gender	Gait Speed	Physical Issues
1	80	M	0.615 m/s ± 0.0096 m/s	Visual impairment; osteoarthritis; low back pain
2	78	F	0.654 m/s ± 0.0109 m/s	Osteoarthritis (right shoulder and leg); spinal discs herniation
3	85	F	0.607 m/s ± 0.0094 m/s	Meniscus surgery in both knees
4	87	M	0.498 m/s ± 0.0063 m/s	Osteoarthritis (left knee)
5	86	M	0.687 m/s ± 0.0120 m/s	Heart surgery. Lower limbs weakness
6	91	M	0.597 m/s ± 0.0090 m/s	Vestibular disorder
7	76	M	0.763 m/s ± 0.0148 m/s	Visual impairment; low back pain
8	74	M	0.792 m/s ± 0.0160 m/s	Right knee prosthesis
9	86	M	0.749 m/s ± 0.0143 m/s	Osteoarthritis; low back pain; left meniscus surgery
10	94	M	0.563 m/s ± 0.0080 m/s	Anemia; pacemaker; left knee pain
